# Insights into the Geographical Origins of the Cabo Verde Green Monkey

**DOI:** 10.3390/genes15040504

**Published:** 2024-04-17

**Authors:** Lara Almeida, Ivo Colmonero-Costeira, Maria J. Ferreira da Silva, Cecilia Veracini, Raquel Vasconcelos

**Affiliations:** 1 CIBIO, Centro de Investigação em Biodiversidade e Recursos Genéticos, InBIO Laboratório Associado, Campus de Vairão, Universidade do Porto, 4485-661 Vairão, Portugal; 2 Departamento de Biologia, Faculdade de Ciências, Universidade do Porto, 4169-007 Porto, Portugal; 3 BIOPOLIS Program in Genomics, Biodiversity and Land Planning, CIBIO, Campus de Vairão, 4485-661 Vairão, Portugal; 4 Organisms and Environment Division, School of Biosciences, Sir Martin Evans Building, Museum Avenue, Cardiff CF10 3AX, Wales, UK; 5 CIAS, Department of Life Sciences, University of Coimbra, 3000-456 Coimbra, Portugal; 6 Centre of Public and Political Administration, Institute of Social and Political Sciences, University of Lisbon, 1300-663 Lisboa, Portugal

**Keywords:** African Cercopithecids, exotic species, Macaronesia, non-human primate biogeography, wildlife trade

## Abstract

The green monkey *Chlorocebus sabaeus*, L. 1766, native to West Africa, was introduced to the Cabo Verde Archipelago in the 16th century. Historical sources suggest that, due to the importance of Cabo Verde as a commercial entrepôt in the Atlantic slave trade, establishing the precise place of origin of this introduced species is challenging. Non-invasive fecal samples were collected from feral and captive green monkey individuals in Cabo Verde. Two mitochondrial fragments, HVRI and cyt *b*, were used to confirm the taxonomic identification of the species and to tentatively determine the geographic origin of introduction to the archipelago from the African continent. By comparing the new sequences of this study to previously published ones, it was shown that Cabo Verde individuals have unique haplotypes in the HVRI, while also showing affinities to several populations from north-western coastal Africa in the cyt *b*, suggesting probable multiple sources of introduction and an undetermined most probable origin. The latter is consistent with historical information, but may also have resulted from solely using mtDNA as a genetic marker and the dispersal characteristics of the species. The limitations of the methodology are discussed and future directions of research are suggested.

## 1. Introduction

Various species of domestic and wild mammals were introduced to the Atlantic islands during the European expansion of the Modern period [1,2]. The Cabo Verde Islands is one oceanic Macaronesian archipelagos in the North Atlantic circa 500 km in front of the coast of Senegal that experienced similar transformations [3]. It is composed of 10 main islands originally inhabited solely by bats [4]. These introductions took place in approximately 1462 when, as contemporary sources attest, the islands began to be systematically populated, serving as a strategic stop for Atlantic navigation and the slave trade from Africa [5]. In this and subsequent periods, the Cabo Verde Archipelago has been the target of introductions of various wild mammal species, including the house mouse *Mus musculus* L. 1758, the brown rat *Rattus norvegicus* Berkenhout 1769, the black rat *Rattus rattus* L. 1758, the rabbit *Oryctolagus cuniculus* L. 1758, and the green monkey *Chlorocebus sabaeus*, L. 1766 [2].

Green monkeys are native to West Africa, distributed from Senegal, Gambia, and Guinea-Bissau to the western part of the Volta River system in Ghana [6]. This medium-sized species can inhabit a wide range of habitats, including the *Acacia* savannah, coastal forest, marginal parts of lowland tropical rainforest, mangroves, gallery forests, and cultivated areas [7]. Its diet is based on a diverse range of leaves, fruits, animal prey, gum, flowers, and seeds, which may vary according to location and season [8]. Green monkeys are primarily terrestrial and live in complex social groups ranging from 18 to 33 individuals [6]. Historical sources suggest that they were introduced in Cabo Verde on Santiago Island in the late 16th century or even earlier [9]. Currently, wild populations of this species are found on the islands of Santiago and Brava [10].

Species of this genus are easily bred in captivity due to their omnivorous diet and their medium size makes them easy to transport. These animals have been used as pets or entertainment animals since the Bronze Age in various cultures and civilizations [11,12]. During the Age of Exploration, primates were predominantly kept as pets and sold at high prices in Europe [13]. *Chlorocebus* specimens were imported in great numbers to renaissance Europe, featured in artworks of the period, and described in 16th-century natural treatises [13]. Monkeys could become a source of food on long journeys and it is thought that this might have been one of the reasons for the introduction of the green monkey to Cabo Verde and other Atlantic islands along the slave route, such as Barbados, Nevis, Saint Martin, and Saint Kitts in the West Indies [1,14].

Previous studies have analysed the history of the origin and presence of wild populations of these animals in Cabo Verde [10]. They found that it is difficult to establish the exact moment of their introduction and their place of origin. Cabo Verdean monkeys may originate from different populations dispersed in West Africa such as the former Portuguese Guinea (Guinea-Bissau, which until the mid-19th century included present-day southern Senegal). The most significant activity carried out in these regions by the Portuguese and other Europeans was the slave trade and Santiago Island was the principal port of arrival and the centre of the slave trade in the archipelago [15]. The slave trade occurred in a few main regions: the Gambia River, the Cacheu region in present-day Guinea-Bissau, and the Cape Verde peninsula, near the present-day capital of Senegal, Dakar [15]. In addition, since the 16th century, merchants from Santiago used to venture to more southerly regions, including present-day French Guiana, reaching as far as Sierra Leone [15]. Furthermore, we cannot exclude the role of the French, who, since the 16th century, were important agents in the slave trade in Senegambia and the Bight of Benin, maintaining a strong relationship with the Santiago Islands. French navigators traded slaves from Senegal to the West Indian Islands [16] and they may be responsible for the introduction of *C. sabaeus* to the islands of St. Kitts in the 17th century [1]. Green monkeys may have been transported to Santiago numerous times due to the island’s significance as the commercial centre of the archipelago for several centuries, although its economy and fortunes experienced various fluctuations in relation to changes in the world economy [15]. In more recent periods, historical sources indicate that the trade of monkeys continued in various forms and there were instances when monkeys managed to escape from captivity. For example, in the second half of the 19th century, about 20 monkeys were brought to Santiago from the former Portuguese Guinea and escaped from their cages during disembarkation at Praia, Santiago Island, and at Furna, Brava Island [17,18]. Therefore, we can postulate that the populations of green monkeys on Santiago and Brava islands may have resulted from multiple introductions and that the localities of origin may have been diverse rather than only from Guinea-Bissau as some authors claim [18]. This consideration is especially pertinent, given that these animals could be sold in places far from their locality of capture, being commonly used as pets.

Genetic information can be used to identify the geographic source of an introduced population on islands (e.g., [19]). For that, the researcher must gather information on the genetic variation of the regional metapopulation and how the variation is structured across space and use statistical approaches to identify close genetic relationships between the geographically-unassigned individuals and samples of known geographic origin, i.e., the reference dataset [20]. Molecular methods have been particularly useful to investigate, for instance, the source population of individuals kept in zoos (e.g., chimpanzees, *Pan troglodytes* ssp.) [20]. The accuracy of the DNA-based spatial assignment correlates to the ability to identify geographically-specific genetic variation, which in turn is dependent on the genetic markers used and the dimension and variability of reference datasets [21].

Past studies investigated the geographic origin of introduced green monkeys in the West Indies (Barbados, St. Kitts, and Nevis islands) using molecular-based spatial assignment methods. According to previous historical studies [14], the West Indian monkeys may have heterogenous origins coming either directly from Senegal and Gambia (St. Kitts population), from the Gold Coast (Barbados population), or even from specimens of Cabo Verdean origins. The molecular species identification of one individual from the St. Kitts island was compatible with a West Africa origin and the species assignment to *C. sabaeus* (previously named *Cercopithecus aethiops sabaeus* [22]). Previous studies re-analysed the same sample to identify its most likely country of origin in a geographically broader analysis of the genus *Chlorocebus* and it clustered together with other samples from Senegal and Mauritania [23]. Other authors [24] studied the introduced *Chlorocebus* population in the south-eastern United States and suggested that data align a West Africa origin of the *C. sabaeus* specimen, with identical haplotypes to the ones found in Senegal.

The objective of this study is to enhance the understanding of the origin and molecular species identification of Cabo Verde green monkeys using non-invasive mitochondrial DNA samples and available geo-referenced datasets from mainland Africa and the Americas. It is hypothesised that most of these animals originate from ancient Senegambia and Portuguese Guinea, with further contributions from individuals from other regions within the geographical range of *C. sabaeus.*

## 2. Materials and Methods

### 2.1. Data Collection

**Figure 1:**
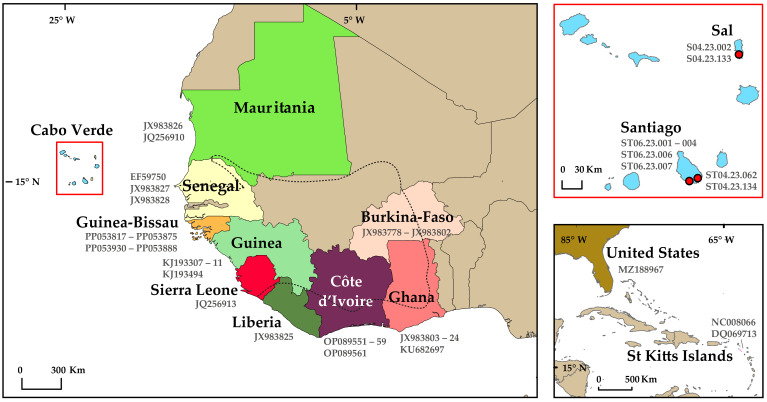
Maps representing the geographical location of the samples used in this study for HVRI and cyt *b* mitochondrial DNA markers. The left corner represents samples from mainland Africa (the dashed line represents the distribution range of *Chlorocebus. sabaeus*, adapted from the International Union for Conservation of Nature (IUCN) Red List data [25]), the upper right corner samples from Cabo Verde (enlarged in the red box; their locations marked with red dots), and samples from the Americas on the bottom right. Samples retrieved from the GenBank are represented by accession numbers and samples from this study are represented by tissue codes and their locations are marked with red dots. Country colours correspond to the haplotype colours in Figure 3. Check Table S1 for further details.

**Figure 2:**
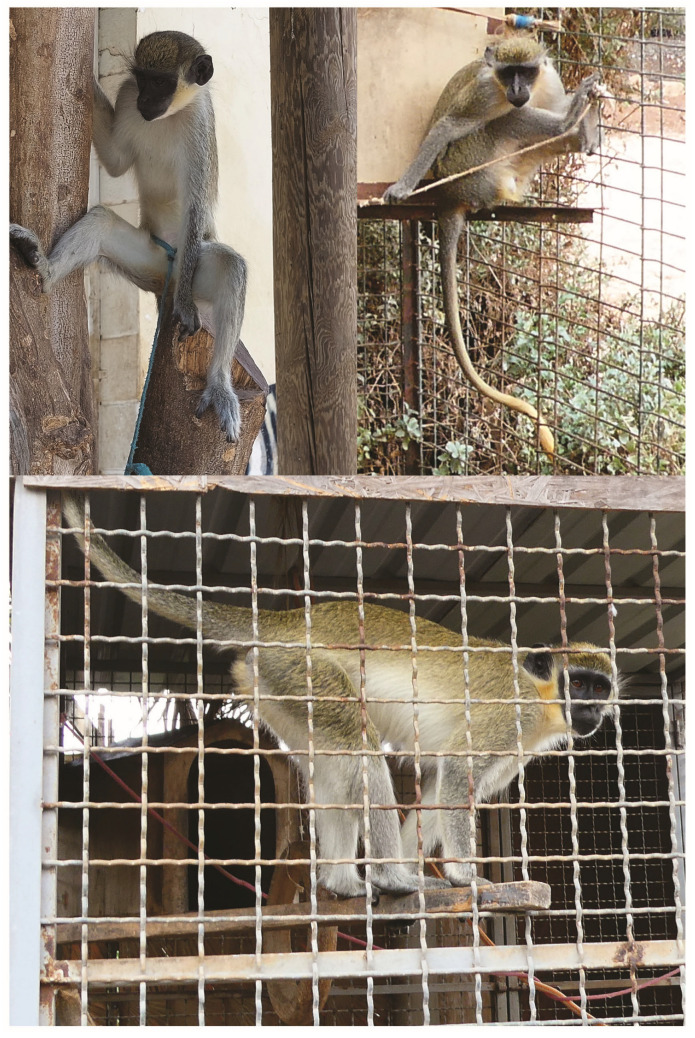
Pictures of some of the captive green monkeys sampled for this study. The upper left corner represents the privately owned adult female from Cidade Velha, Santiago (ST.06.23.002; photo by Cecilia Veracini). The upper right corner and bottom pictures represent, respectively, the adult male and female from Santa Maria, Sal (S04.23.133 and S04.23.002; photos by Raquel Vasconcelos).

Biological material was collected on the islands of Sal and Santiago, in Cabo Verde (Figure 1; Table S1). Fecal samples were collected non-invasively from wild animals near water lines in São Filipe (two samples) and captive animals from Santiago and Sal (10 samples). The captive specimens (Figure 2) were kept in the rescuing center ‘Santuario dos Macacos’ (seven samples) and were owned by a local (one sample), all in Cidade Velha, Santiago, and in the ‘Pachamama Ecopark’ near Santa Maria, Sal (two samples). All samples were collected from different individuals.

About 2 mL of faecal pellet was scrapped from the outside of each sample and preserved at room temperature in 15-mL falcon tubes filled up with 96% ethanol until DNA extraction. Gloves were used during collection to prevent contamination. The collection of faecal samples was approved by local authorities (Nº 021 and 028/DNA/2023). No animals were handled to obtain the samples, ensuring their welfare throughout the study.

DNA was extracted using the Magmax Core Kit from Applied Biosystems on the extraction robot (KingFisher Apex), following the manufacturer’s instructions conducted in a laboratory dedicated to the extraction of non-invasive DNA. To proceed with the molecular identification of the species and the investigation of the putative geographic origin of the Cabo Verde population, two regions of mitochondrial DNA were used as genetic markers and amplified by Polymerase Chain Reaction (PCR): a 329 base pairs (bp) fragment in the HVRI region, using the primers LCERCOHVRI and HCERCOHVRI [19], and a 402 bp fragment of the cytochrome *b* gene (cyt *b*), using the primers GVL14724 and H15149 [26]. These specific mtDNA fragments were chosen because they (i) are easily amplified from non-invasive DNA samples and can be used to identify the samples to the species level (e.g., [19]), (ii) have been previously used by other researchers studying wild populations of the green monkey in Guinea-Bissau, which is considered one putative source of the Cabo Verde population (e.g., [19,27]), and (iii) have been extensively used to build geo-referenced datasets for samples collected in the species range (e.g., [26]) and are available in public repositories of genetic data (e.g., GenBank). Please note that, to the best of our knowledge, equivalently large geo-referenced datasets based on nuclear or Y-linked loci are not available.

The PCR started with an initial activation temperature of 95 °C for 15 min, a denaturation temperature of 94 °C for 30 s, annealing at 60 °C for 30 s, and extension at 72 °C for 30 s for 39 cycles and 15 min at 72° for both genes. The successfully amplified PCR products from both markers were sequenced bidirectionally using Sanger technology (AB3500XL, Applied Biosystems, Massachusetts, MA, USA) at CIBIO facilities and made available on GenBank (PP620289–PP620302). Chromatograms of forward and reverse sequences were checked by eye for quality and sequences were concatenated using Geneious Prime 2023.2.1 [28]. Consensus sequences were identified at the specific level using BLAST [29].

### 2.2. Geo-Referenced mtDNA Dataset of Reference and Network Analyses

BLAST was used to search for the most similar mtDNA sequences available on GenBank to the new sequences of this study (ID from 80 to 100%) of which the geographic origin was gathered into a dataset of reference (i.e., country and, when possible, the site within the country). Of the mtDNA regions of interest, a total of 72 sequences from seven countries with 262 bp of HVRI and 141 sequences from 12 countries with 345 bp of cyt *b* were used to evaluate the genetic proximity between Cabo Verdean green monkey populations and other populations in West Africa and that were introduced in the Americas to determine their most likely geographic origin. Detailed information on the locations and GenBank accession codes can be found in Figure 1 and Table S1. A MAFFT alignment using the new sequences and the reference dataset was built using Geneious [30]. Then, haplotype networks were constructed for each fragment to visualise the relationships between all the populations using TCS [31] with default parameters. Finally, tcsBU [32] was used to visualise the results.

## 3. Results

### 3.1. DNA Extraction and Amplification

Whole-genomic DNA present in 10 of the 12 pellets was successfully extracted and amplified for either one or both fragments (two from the island of Sal and eight from Santiago; Figure 1). In total, 14 sequences were successfully amplified: a set of 7 sequences for cyt *b* and another set of 7 for HVRI, as two samples failed to amplify to both fragments and six different samples to one of the two fragments. Those sequences presented between 100% to 98% ID as *C. sabaeus* using BLAST.

### 3.2. mtDNA Variation

For the Cabo Verde samples, two unique haplotypes for the HVRI fragment and three distinct haplotypes for the cyt *b* fragment were obtained.

**Figure 3:**
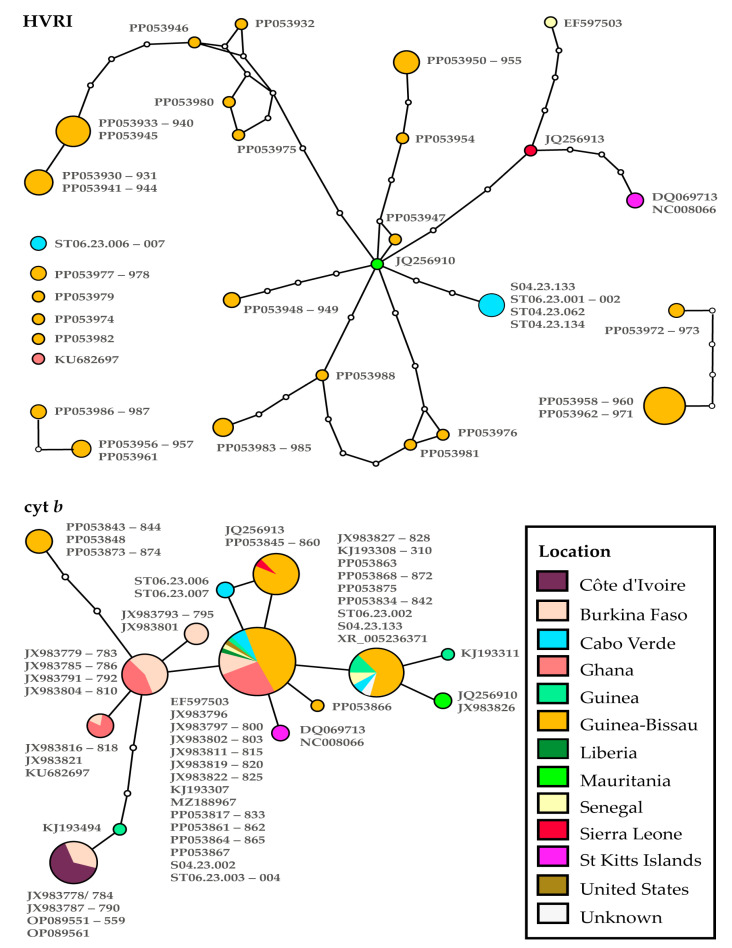
Haplotype network for the mitochondrial marker HVRI (329 bp; **top**) and cyt *b* (402 bp; **bottom**). The lines represent mutational steps, the circles represent haplotypes, and the dots represent missing haplotypes. The haplotypes are geographically coded by colours that correspond to the colours of the countries shown in Figure 1. The size of the circles represents the frequency of the haplotype. Check Table S1 for further details.

The networks built using HVRI and cyt *b* fragments in Figure 3 show the relationships of *C. sabaeus* haplotypes found in Cabo Verde with wild populations in mainland Africa and other populations introduced in the Americas, in St. Kitts Islands, Florida, and an unknown location [24,33] (three samples; for locations and references, please check Table S1).

Considering a 95% parsimony connection limit, one main network and two smaller ones were built using HVRI sequences (Figure 2). Ten of the haplotypes were left out of the main network (from Guinea-Bissau, Ghana, and Cabo Verde). Six of them were formed exclusively by Guinea-Bissau haplotypes sampled across the southern part of the country at Cufada Lagoons Natural Park (CLNP), Dulombi National Park (DNP), Boé National Park (BNP), and Cantanhez National Park (CNP). Three unique haplotypes left out of the main network were recovered from CLNP: one from DNP and two isolated networks with unique haplotypes from DNP and BNP as well as CFNP and BNP, separated by two and five mutation steps, respectively. One unique haplotype left out of the main network was from Ghana and another was from Cabo Verde. The main network presented haplotypes from five African countries and the introduced American populations were divided into six subgroups with some geographical structure, with one to five mutation steps from the central Mauritanian haplotype. Of those, four presented haplotypes only from Guinea-Bissau (two with unique haplotypes from BNP, one with individuals from Bijagós Islands, and another with individuals from DNP and CLNP); one presented haplotypes from Sierra Leone, Senegal, and St Kitts Islands; and the sixth presented haplotypes exclusive from Cabo Verde. Hence, of the two unique haplotypes sampled in Cabo Verde sequenced for the HVRI fragment, one is closely related to the Mauritanian central haplotype (three mutations apart) and the other haplotype is non-connected to any haplogroup in the main network.

In the cyt *b* haplotype network, 14 different haplotypes were identified, all connected using a 95% parsimony connection limit. In this network, three subgroups can be distinguished: one comprising a large number of haplotypes from regions closer to the Gulf of Guinea (such as Côte d’Ivoire, Burkina Faso, and Guinea) located in the south of the species range, another comprising haplotypes exclusive to Guinea-Bissau from DNP and BNP, and a third comprising haplotypes from north-western African countries (at the northern part of the species distribution) and St Kitts and Cabo Verde islands. Each of the first two subgroups is separated from the third by at least three mutational steps (Figure 3).

Of the three distinct haplotypes sampled in Cabo Verde for the cyt *b* fragment, one is unique and two are identical to haplotypes sampled in mainland African countries and present over a large geographic area. One is indistinguishable from a central and very frequent haplotype, which is also present in Ghana, Liberia, Burkina Faso, Senegal, the United States, and Guinea-Bissau. The other haplotype is only one mutation step from the central haplotype and is shared among Guinea-Bissau, Senegal, and Guinea haplotypes. The unique haplotype found in Cabo Verde is one mutation step from the central haplotype and one mutation step from Guinea-Bissau (from BNP and CFNP) and Sierra Leone samples.

## 4. Discussion

In this work, the geographic origin in mainland West Africa of the green monkey population introduced in Cabo Verde was investigated using molecular spatial assignment tools. For the first time, mtDNA haplotypes of the green monkey of Cabo Verde Islands were described using non-invasive faecal DNA. Results showed two Cabo Verdean haplotypes for the HVRI fragment and three haplotypes for the cyt *b* fragment, which are identical or genetically similar to haplotypes sampled in Western mainland Africa in countries such as Ghana, Liberia, Burkina Faso, Senegal, Guinea and Guinea-Bissau. However, the analyses were largely inconclusive in assigning one or few single probable(s) origin(s). These results can be explained by (i) an heterogenous (multiple sources) origin of the introduced population in Cabo Verde and/or (ii) limitations posed by the sole use of mtDNA as a genetic marker and dispersal features of the species.

The probable multiple sources of Cabo Verde green monkeys align with historical evidence and support our hypothesis of multiple introductions and geographical origins [16,17,18]. This study suggests six countries as possible sources of the introduced green monkey in Cabo Verde for the first time but, due to methodological limitations (see below), it was not possible to rank these distinct locations as the most probable origin.

The two mtDNA haplotype networks were built using a large reference dataset (213 geo-referenced mtDNA sequences), suggesting a considerable lack of spatial structure of genetic variation for the green monkey across the species range in West Africa. In particular, the two networks displayed haplogroups formed by haplotypes sampled in various and, for some cases, distant locations (e.g., cyt *b* central haplotype comprised samples with geographic distances of over 1500 km). This result of a lack of geographic structure for mtDNA variation is similar to what was found at a smaller scale in Guinea-Bissau, where two significantly divergent haplogroups that were estimated to have diverged 1.2 million years ago were found in locations distancing only 150 km apart [27] and thus should reflect a feature of the species instead of sampling biases.

The lack of geographic structure in mtDNA variation may be related to female dispersal events for *C. sabaeus*. In social mammals, such as primates, dispersal may be mediated by both sexes (i.e., both males and females leave their natal group when reaching sexual maturity to reproduce elsewhere) or the behaviour can be displayed by one sex in a greater extent (i.e., sex-biased dispersal) [6]. In such cases, individuals of the dispersing sex tend to emigrate to a different group whereas the individuals of the philopatric sex remain in the natal group [34,35]. The trait of which sex tends to disperse is extremely conserved among closely related primates or populations of the same species [36], even considering populations facing extreme environmental variation [37] thought to have been selected during the species’ evolutionary history. The great majority of Cercopithecine primates display a male-biased dispersal pattern [38], although female-biased dispersal species have been identified (e.g., the Guinea baboons, *Papio papio*, [37]. In the case of *C. sabaeus*, males are expected to be the most frequently dispersing sex but transference of females between social groups has been described [39]. The species’ dispersal patterns affect population’s genetic structure [40]. Specifically, when dispersal is carried out exclusively or to a significantly greater extent by only one sex, it is predicted that there are contrasting patterns of genetic structure between the nuclear genome (transmitted by the maternal and paternal lines) and that the genome is inherited through the philopatric sex (i.e., the mitochondrial genome for females and the Y chromosome for males [40]). For male-biased dispersal species, mtDNA variation is expected to be strongly substructured across space and autosomal or Y-linked variation are anticipated to have little or no geographic structure [41]. By contrast, in species with a degree of female dispersal (regardless of males remaining philopatric), both mtDNA and autosomal variation are shuffled across space by female-mediated gene flow and a comparable substructure is anticipated for the two genomes [40]. Female-biased dispersal between closely located groups over numerous generations may lead to a pattern in which (i) mtDNA variation is uncorrelated to the geographic location of demes, (ii) genetically differentiated haplotypes are found in the same or geographically close demes, and (iii) haplotypes are shared by many and distant demes (over 500 km for some haplotypes, e.g., the Guinea baboon [37]). A spatial pattern of mtDNA variation, in which identical haplotypes are shared between distant countries, resembles what we found here for the green monkey using available mtDNA datasets and limits our understanding of the origin and process of introduction of the population in Cabo Verde. To the best of our knowledge, the only study investigating the species phylogeographic structure in West Africa was carried out more than 10 years ago. The authors found that individuals sampled in Senegal and Mauritania are genetically distinct from the ones in Ghana and Burkina Faso but have not analysed mtDNA variation substructure in greater detail [23].

## 5. Conclusions

This work suggests multiple sources of Cabo Verde green monkeys and six countries as possible sources. It also highlights that in species with a degree of female-mediated geneflow and in which mtDNA variation has low geographic specificity, the efficiency of mtDNA-based spatial assignment methods of samples of unknown origin is compromised. By contrast to what was found here for the green monkey, mtDNA-based spatial assignment of ivory products from the African elephant *Loxodonta africana* is highly efficient because females are almost strictly philopatric and mtDNA exhibits high geographic specificity [24]. In the African elephant, mtDNA clades tend to have restricted geographic distributions (and may be country-specific) [21] and mtDNA markers may be used with great efficiency to identify the provenience of geographically unassigned samples.

To address the limitations of this work, future work should use variable autosomal and Y chromosome-linked loci (not yet available), in combination with mtDNA data, to investigate the geographic origin of introduced populations of green monkeys in Atlantic islands, which could be developed using genome-wide data from wild individuals [27] and searching algorithms used in closely-related Cercopithecine species (see [42]).

## Data Availability

The sequences generated in this article are available in the GenBank database (accession codes PP620289–PP620302).

## Supplementary Materials

The following supporting information can be downloaded at https://www.mdpi.com/article/10.3390/genes15040504/s1. Table S1: Details of the green monkey samples used in this study. The tissue code or GenBank accession number (GenBank), the amplified gene, the country, the locality (NP, National Park), geographical coordinates (Lat, latitude; Long, longitude) and their origin (W, wild; M, museum; C, captive), and published references (Ref.) are indicated (*, this study); File S1: Portuguese version of the abstract. References [43,44,45,46] are cited in the Supplementary Materials.
