# Supplementary material for: Insights into the Geographical Origins of the Cabo Verde Green Monkey
Source: Genes (Basel). 2024 Apr 17;15(4):504. doi: 10.3390/genes15040504 (PMC11050204; doi:10.3390/genes15040504)
Supplement: Supplementary file 1 [file genes-15-00504-s001.zip › genes-2916591-supplementary.pdf]

Table S1. Details of the green monkey samples used in this study. The tissue code or GenBank accession number (GenBank), the amplified gene, and the country, locality (NP, National Park), and geographical coordinates (Lat, latitude; Long, longitude), and their origin (W, wild; M, museum; C, captive), and published references (Ref.) are indicated (*, this study).

GenBank	Gene	Country	Locality	Lat	Long	Origin	Ref.
JX983778	cyt *b*	Burkina Faso	Comoe Leraba	9.902600	-4.654540	W	[23]
JX983779	cyt *b*	Burkina Faso	Comoe Leraba	9.902600	-4.654540	W	[23]
JX983780	cyt *b*	Burkina Faso	Comoe Leraba	9.902600	-4.654540	W	[23]
JX983781	cyt *b*	Burkina Faso	Comoe Leraba	9.848960	-4.623020	W	[23]
JX983782	cyt *b*	Burkina Faso	Comoe Leraba	9.848960	-4.623020	W	[23]
JX983783	cyt *b*	Burkina Faso	Comoe Leraba	9.848960	-4.623020	W	[23]
JX983784	cyt *b*	Burkina Faso	Comoe Leraba	9.852910	-4.614150	W	[23]
JX983785	cyt *b*	Burkina Faso	Comoe Leraba	9.869920	-4.656260	W	[23]
JX983786	cyt *b*	Burkina Faso	Comoe Leraba	9.869920	-4.656260	W	[23]
JX983787	cyt *b*	Burkina Faso	Comoe Leraba	9.862070	-4.669740	W	[23]
JX983788	cyt *b*	Burkina Faso	Comoe Leraba	9.862070	-4.669740	W	[23]
JX983789	cyt *b*	Burkina Faso	Comoe Leraba	9.862070	-4.669740	W	[23]
JX983790	cyt *b*	Burkina Faso	Comoe Leraba	9.862070	-4.669740	W	[23]
JX983791	cyt *b*	Burkina Faso	Comoe Leraba	9.775300	-4.602710	W	[23]
JX983792	cyt *b*	Burkina Faso	FC Deux Bale	11.550790	-2.957570	W	[23]
JX983793	cyt *b*	Burkina Faso	Ranch Nazinga	11.161820	-1.609670	W	[23]
JX983794	cyt *b*	Burkina Faso	Ranch Nazinga	11.135140	-1.612650	W	[23]
JX983795	cyt *b*	Burkina Faso	Ranch Nazinga	11.155940	-1.614450	W	[23]
JX983796	cyt *b*	Burkina Faso	Ranch Nazinga	11.148700	-1.623340	W	[23]
JX983797	cyt *b*	Burkina Faso	Ranch Nazinga	11.148700	-1.623340	W	[23]
JX983798	cyt *b*	Burkina Faso	Ranch Nazinga	11.148700	-1.623340	W	[23]
JX983799	cyt *b*	Burkina Faso	Ranch Nazinga	11.148700	-1.623340	W	[23]
JX983800	cyt *b*	Burkina Faso	Ranch Nazinga	11.155470	-1.610300	W	[23]
JX983801	cyt *b*	Burkina Faso	Ranch Nazinga	11.155470	-1.610300	W	[23]
JX983802	cyt *b*	Burkina Faso	Ranch Nazinga	11.155470	-1.610300	W	[23]
JX983803	cyt *b*	Ghana	Kratshi/Krachi	7.800000	-0.050000	W	[23]
JX983804	cyt *b*	Ghana	Bui NP	8.290830	-2.284650	W	[23]
JX983805	cyt *b*	Ghana	Bui NP	8.291140	-2.284310	W	[23]
JX983806	cyt *b*	Ghana	Bui NP	8.291140	-2.284310	W	[23]
JX983807	cyt *b*	Ghana	Bui NP	8.290900	-2.283880	W	[23]
JX983808	cyt *b*	Ghana	Bui NP	8.291260	-2.283150	W	[23]
JX983809	cyt *b*	Ghana	Bui NP	8.290830	-2.284650	W	[23]
JX983810	cyt *b*	Ghana	Bui NP	8.290830	-2.284650	W	[23]
JX983811	cyt *b*	Ghana	Mole NP	9.260080	-1.860580	W	[23]
JX983812	cyt *b*	Ghana	Mole NP	9.260580	-1.861470	W	[23]
JX983813	cyt *b*	Ghana	Mole NP	9.258760	-1.847140	W	[23]
JX983814	cyt *b*	Ghana	Mole NP	9.258760	-1.847140	W	[23]
JX983815	cyt *b*	Ghana	Mole NP	9.258760	-1.847140	W	[23]
JX983816	cyt *b*	Ghana	Mole NP	9.251830	-1.861050	W	[23]

JX983817	cyt *b*	Ghana	Mole NP	9.251830	-1.861050	W	[23]
JX983818	cyt *b*	Ghana	Mole NP	9.251830	-1.861050	W	[23]
JX983819	cyt *b*	Ghana	Mole NP	9.251830	-1.861050	W	[23]
JX983820	cyt *b*	Ghana	Mole NP	9.251830	-1.861050	W	[23]
JX983821	cyt *b*	Ghana	Mole NP	9.251830	-1.861050	W	[23]
JX983822	cyt *b*	Ghana	Mole NP	9.251830	-1.861050	W	[23]
JX983823	cyt *b*	Ghana	Shai Hills RR	5.897770	0.068970	W	[23]
JX983824	cyt *b*	Ghana	Shai Hills RR	5.890010	0.043820	W	[23]
JX983825	cyt *b*	Liberia	Gola Country	7.443170	-10.777790	C	[23]
JX983826	cyt *b*	Mauritania	Podor, Senegal	16.548440	-14.243040	M	[23]
JX983827	cyt *b*	Senegal	Niokolo Koba NP	13.075360	-12.722390	W	[23]
JX983828	cyt *b*	Senegal	Niokolo Koba NP	13.025770	-13.237360	W	[23]
KJ193307	cyt *b*	Guinea	Koubia	11.780000	-11.790000	W	[26]
KJ193308	cyt *b*	Guinea	Koubia	11.780000	-11.790000	W	[26]
KJ193309	cyt *b*	Guinea	Koubia	11.780000	-11.790000	W	[26]
KJ193310	cyt *b*	Guinea	Koubia	11.780000	-11.790000	W	[26]
KJ193311	cyt *b*	Guinea	Koubia	11.780000	-11.790000	W	[26]
KJ193494	cyt *b*	Guinea	Koubia	11.780001	-11.790001	W	[26]
EF597503	cyt *b*	Senegal	-	-	-	C	[43]
JQ256910	cyt *b*	Mauritania	Podor, Senegal	16.548440	-14.243040	M	[44]
JQ256913	cyt *b*	Sierra Leone	-	-	-	M	[44]
XR 005236371	cyt *b*	-	-	-	-	C	-
NC 008066	cyt *b*	St Kitts Island	VMRC	-	-	C	[33]
DQ069713	cyt *b*	St Kitts Island	VMRC	-	-	C	[33]
KU682697	cyt *b*	Ghana	-	-	-	W	[45]
MZ188967	cyt *b*	United States	Dania Beach	-	-	W	[24]
OP089551	cyt *b*	Côte d'Ivoire	Yopougon	5.317666	-4.089991	W	[46]
OP089552	cyt *b*	Côte d'Ivoire	Yopougon	5.317666	-4.089991	W	[46]
OP089553	cyt *b*	Côte d'Ivoire	Yopougon	5.317666	-4.089991	W	[46]
OP089554	cyt *b*	Côte d'Ivoire	Yopougon	5.317666	-4.089991	W	[46]
OP089555	cyt *b*	Côte d'Ivoire	Yopougon	5.317666	-4.089991	W	[46]
OP089556	cyt *b*	Côte d'Ivoire	Yopougon	5.317666	-4.089991	W	[46]
OP089557	cyt *b*	Côte d'Ivoire	Yopougon	5.317666	-4.089991	W	[46]
OP089558	cyt *b*	Côte d'Ivoire	Yopougon	5.317666	-4.089991	W	[46]
OP089559	cyt *b*	Côte d'Ivoire	Yopougon	5.317666	-4.089991	W	[46]
OP089561	cyt *b*	Côte d'Ivoire	Yopougon	5.317666	-4.089991	W	[46]
PP620289	cyt *b*	Cabo Verde	Sal	16.62153	-22.91789	C	*
PP620292	cyt *b*	Cabo Verde	Sal	16.62153	-22.91789	C	*
PP620290	cyt *b*	Cabo Verde	Santiago	14.91666	-23.60416	C	*
PP620291	cyt *b*	Cabo Verde	Santiago	14.91666	-23.60416	C	*
PP620293	cyt *b*	Cabo Verde	Santiago	14.91666	-23.60416	C	*
PP620294	cyt *b*	Cabo Verde	Santiago	14.91666	-23.60416	C	*
PP620295	cyt *b*	Cabo Verde	Santiago	14.91666	-23.60416	C	*
PP053817	cyt *b*	Guinea-Bissau	Bijagós	11.181079	-16.014061	W	[27]
PP053818	cyt *b*	Guinea-Bissau	Bijagós	11.181213	-16.014040	W	[27]

PP053819	cyt *b*	Guinea-Bissau	Bijagós	11.156381	-16.023569	W	[27]
PP053820	cyt *b*	Guinea-Bissau	Bijagós	11.142019	-16.023033	W	[27]
PP053821	cyt *b*	Guinea-Bissau	Bijagós	11.142610	-16.024138	W	[27]
PP053822	cyt *b*	Guinea-Bissau	Bijagós	11.189673	-16.014547	W	[27]
PP053823	cyt *b*	Guinea-Bissau	Bijagós	11.189663	-16.014599	W	[27]
PP053824	cyt *b*	Guinea-Bissau	Bijagós	11.147685	-16.024084	W	[27]
PP053825	cyt *b*	Guinea-Bissau	Bijagós	11.149734	-16.020313	W	[27]
PP053826	cyt *b*	Guinea-Bissau	Bijagós	11.156542	-16.019891	W	[27]
PP053827	cyt *b*	Guinea-Bissau	Bijagós	11.156617	-16.019783	W	[27]
PP053828	cyt *b*	Guinea-Bissau	Bijagós	11.178124	-16.017105	W	[27]
PP053829	cyt *b*	Guinea-Bissau	Bijagós	11.177919	-16.017429	W	[27]
PP053830	cyt *b*	Guinea-Bissau	Bijagós	11.177932	-16.017398	W	[27]
PP053831	cyt *b*	Guinea-Bissau	Bijagós	11.177731	-16.017533	W	[27]
PP053832	cyt *b*	Guinea-Bissau	Bijagós	11.084400	-16.221100	W	[27]
PP053833	cyt *b*	Guinea-Bissau	Bijagós	11.271042	-16.174266	W	[27]
PP053834	cyt *b*	Guinea-Bissau	Boé NP	11.832394	-13.805349	W	[27]
PP053835	cyt *b*	Guinea-Bissau	Boé NP	11.932757	-13.785056	W	[27]
PP053836	cyt *b*	Guinea-Bissau	Boé NP	11.938790	-13.781128	W	[27]
PP053837	cyt *b*	Guinea-Bissau	Boé NP	11.927518	-14.238246	W	[27]
PP053838	cyt *b*	Guinea-Bissau	Boé NP	11.927528	-14.238303	W	[27]
PP053839	cyt *b*	Guinea-Bissau	Boé NP	11.927584	-14.238426	W	[27]
PP053840	cyt *b*	Guinea-Bissau	Boé NP	11.927579	-14.238382	W	[27]
PP053841	cyt *b*	Guinea-Bissau	Boé NP	11.920890	-14.239909	W	[27]
PP053842	cyt *b*	Guinea-Bissau	Boé NP	11.920981	-14.239945	W	[27]
PP053843	cyt *b*	Guinea-Bissau	Boé NP	11.933605	-13.751766	W	[27]
PP053844	cyt *b*	Guinea-Bissau	Boé NP	11.933219	-13.751964	W	[27]
PP053845	cyt *b*	Guinea-Bissau	Boé NP	11.933229	-13.752008	W	[27]
PP053846	cyt *b*	Guinea-Bissau	Boé NP	11.933180	-13.752026	W	[27]
PP053847	cyt *b*	Guinea-Bissau	Boé NP	11.933180	-13.752026	W	[27]
PP053848	cyt *b*	Guinea-Bissau	Boé NP	11.933241	-13.751948	W	[27]
PP053849	cyt *b*	Guinea-Bissau	Boé NP	11.933191	-13.751988	W	[27]
PP053850	cyt *b*	Guinea-Bissau	Boé NP	11.932444	-13.756259	W	[27]
PP053851	cyt *b*	Guinea-Bissau	Boé NP	11.932556	-13.756233	W	[27]
PP053852	cyt *b*	Guinea-Bissau	Boé NP	11.932493	-13.756258	W	[27]
PP053853	cyt *b*	Guinea-Bissau	Boé NP	11.932562	-13.756249	W	[27]
PP053854	cyt *b*	Guinea-Bissau	Boé NP	11.932574	-13.756227	W	[27]
PP053855	cyt *b*	Guinea-Bissau	Boé NP	11.932548	-13.756235	W	[27]
PP053856	cyt *b*	Guinea-Bissau	Boé NP	11.932437	-13.756303	W	[27]
PP053857	cyt *b*	Guinea-Bissau	Boé NP	11.932504	-13.756295	W	[27]
PP053858	cyt *b*	Guinea-Bissau	Boé NP	11.932544	-13.756337	W	[27]
PP053859	cyt *b*	Guinea-Bissau	Cantanhez	11.111478	-15.128143	W	[27]
PP053860	cyt *b*	Guinea-Bissau	Cantanhez	11.111486	-15.128148	W	[27]
PP053861	cyt *b*	Guinea-Bissau	Cufada Lagoons	11.645079	-15.250188	W	[27]
PP053862	cyt *b*	Guinea-Bissau	Cufada Lagoons	11.645162	-15.250847	W	[27]
PP053863	cyt *b*	Guinea-Bissau	Cufada Lagoons	11.649280	-15.111430	W	[27]

PP053864	cyt *b*	Guinea-Bissau	Cufada Lagoons	11.600576	-15.087729	W	[27]
PP053865	cyt *b*	Guinea-Bissau	Cufada Lagoons	11.600571	-15.087734	W	[27]
PP053866	cyt *b*	Guinea-Bissau	Cufada Lagoons	11.600552	-15.087933	W	[27]
PP053867	cyt *b*	Guinea-Bissau	Cufada Lagoons	11.650172	-15.135876	W	[27]
PP053868	cyt *b*	Guinea-Bissau	Dulombi NP	11.800455	-14.327705	W	[27]
PP053869	cyt *b*	Guinea-Bissau	Dulombi NP	11.812565	-14.396407	W	[27]
PP053870	cyt *b*	Guinea-Bissau	Dulombi NP	11.818034	-14.393708	W	[27]
PP053871	cyt *b*	Guinea-Bissau	Dulombi NP	11.818199	-14.394055	W	[27]
PP053872	cyt *b*	Guinea-Bissau	Dulombi NP	11.818353	-14.394057	W	[27]
PP053873	cyt *b*	Guinea-Bissau	Dulombi NP	11.818437	-14.393795	W	[27]
PP053874	cyt *b*	Guinea-Bissau	Dulombi NP	11.818366	-14.393722	W	[27]
PP053875	cyt *b*	Guinea-Bissau	Dulombi NP	11.818309	-14.393606	W	[27]
JQ256910	HVRI	Mauritania	Podor, Senegal	16.548440	-14.243040	M	[44]
NC 008066	HVRI	St Kitts Island	VMRC	-	-	C	[33]
EF597503	HVRI	Senegal	-	-	-	C	[43]
KU682697	HVRI	Ghana	-	-	-	W	[45]
JQ256913	HVRI	SL, Sierra	-	-	-	M	[44]
DQ069713	HVRI	St Kitts Island	VMRC	-	-	C	[33]
PP620296	HVRI	Cabo Verde	Sal	16.621530	-22.917890	C	*
PP620297	HVRI	Cabo Verde	Santiago	14.975340	-23.517980	W	*
PP620298	HVRI	Cabo Verde	Santiago	14.995420	-23.526960	W	*
PP620299	HVRI	Cabo Verde	Santiago	14.916660	-23.604160	C	*
PP620300	HVRI	Cabo Verde	Santiago	14.916660	-23.604160	C	*
PP620301	HVRI	Cabo Verde	Santiago	14.916660	-23.604160	C	*
PP620302	HVRI	Cabo Verde	Santiago	14.916660	-23.604160	C	*
PP053930	HVRI	Guinea-Bissau	Bijagós	11.181079	-16.014061	W	[27]
PP053931	HVRI	Guinea-Bissau	Bijagós	11.181213	-16.014040	W	[27]
PP053932	HVRI	Guinea-Bissau	Bijagós	11.156381	-16.023569	W	[27]
PP053933	HVRI	Guinea-Bissau	Bijagós	11.142019	-16.023033	W	[27]
PP053934	HVRI	Guinea-Bissau	Bijagós	11.142610	-16.024138	W	[27]
PP053935	HVRI	Guinea-Bissau	Bijagós	11.189673	-16.014547	W	[27]
PP053936	HVRI	Guinea-Bissau	Bijagós	11.189663	-16.014599	W	[27]
PP053937	HVRI	Guinea-Bissau	Bijagós	11.147685	-16.024084	W	[27]
PP053938	HVRI	Guinea-Bissau	Bijagós	11.149734	-16.020313	W	[27]
PP053939	HVRI	Guinea-Bissau	Bijagós	11.156542	-16.019891	W	[27]
PP053940	HVRI	Guinea-Bissau	Bijagós	11.156617	-16.019783	W	[27]
PP053941	HVRI	Guinea-Bissau	Bijagós	11.178124	-16.017105	W	[27]
PP053942	HVRI	Guinea-Bissau	Bijagós	11.177919	-16.017429	W	[27]
PP053943	HVRI	Guinea-Bissau	Bijagós	11.177932	-16.017398	W	[27]
PP053944	HVRI	Guinea-Bissau	Bijagós	11.177731	-16.017533	W	[27]
PP053945	HVRI	Guinea-Bissau	Bijagós	11.084400	-16.221100	W	[27]
PP053946	HVRI	Guinea-Bissau	Bijagós	11.271042	-16.174266	W	[27]
PP053947	HVRI	Guinea-Bissau	Boé NP	11.832394	-13.805349	W	[27]
PP053948	HVRI	Guinea-Bissau	Boé NP	11.932757	-13.785056	W	[27]
PP053949	HVRI	Guinea-Bissau	Boé NP	11.938790	-13.781128	W	[27]

PP053950	HVRI	Guinea-Bissau	Boé NP	11.927518	-14.238246	W	[27]
PP053951	HVRI	Guinea-Bissau	Boé NP	11.927528	-14.238303	W	[27]
PP053952	HVRI	Guinea-Bissau	Boé NP	11.927584	-14.238426	W	[27]
PP053953	HVRI	Guinea-Bissau	Boé NP	11.927579	-14.238382	W	[27]
PP053954	HVRI	Guinea-Bissau	Boé NP	11.920890	-14.239909	W	[27]
PP053955	HVRI	Guinea-Bissau	Boé NP	11.920981	-14.239945	W	[27]
PP053956	HVRI	Guinea-Bissau	Boé NP	11.933605	-13.751766	W	[27]
PP053957	HVRI	Guinea-Bissau	Boé NP	11.933219	-13.751964	W	[27]
PP053958	HVRI	Guinea-Bissau	Boé NP	11.933229	-13.752008	W	[27]
PP053959	HVRI	Guinea-Bissau	Boé NP	11.933180	-13.752026	W	[27]
PP053960	HVRI	Guinea-Bissau	Boé NP	11.933180	-13.752026	W	[27]
PP053961	HVRI	Guinea-Bissau	Boé NP	11.933241	-13.751948	W	[27]
PP053962	HVRI	Guinea-Bissau	Boé NP	11.933191	-13.751988	W	[27]
PP053963	HVRI	Guinea-Bissau	Boé NP	11.932444	-13.756259	W	[27]
PP053964	HVRI	Guinea-Bissau	Boé NP	11.932556	-13.756233	W	[27]
PP053965	HVRI	Guinea-Bissau	Boé NP	11.932493	-13.756258	W	[27]
PP053966	HVRI	Guinea-Bissau	Boé NP	11.932562	-13.756249	W	[27]
PP053967	HVRI	Guinea-Bissau	Boé NP	11.932574	-13.756227	W	[27]
PP053968	HVRI	Guinea-Bissau	Boé NP	11.932548	-13.756235	W	[27]
PP053969	HVRI	Guinea-Bissau	Boé NP	11.932437	-13.756303	W	[27]
PP053970	HVRI	Guinea-Bissau	Boé NP	11.932504	-13.756295	W	[27]
PP053971	HVRI	Guinea-Bissau	Boé NP	11.932544	-13.756337	W	[27]
PP053972	HVRI	Guinea-Bissau	Cantanhez	11.111478	-15.128143	W	[27]
PP053973	HVRI	Guinea-Bissau	Cantanhez	11.111486	-15.128148	W	[27]
PP053974	HVRI	Guinea-Bissau	Cufada Lagoons	11.645079	-15.250188	W	[27]
PP053975	HVRI	Guinea-Bissau	Cufada Lagoons	11.645162	-15.250847	W	[27]
PP053976	HVRI	Guinea-Bissau	Cufada Lagoons	11.649280	-15.111430	W	[27]
PP053977	HVRI	Guinea-Bissau	Cufada Lagoons	11.600576	-15.087729	W	[27]
PP053978	HVRI	Guinea-Bissau	Cufada Lagoons	11.600571	-15.087734	W	[27]
PP053979	HVRI	Guinea-Bissau	Cufada Lagoons	11.600552	-15.087933	W	[27]
PP053980	HVRI	Guinea-Bissau	Cufada Lagoons	11.650172	-15.135876	W	[27]
PP053981	HVRI	Guinea-Bissau	Dulombi NP	11.800455	-14.327705	W	[27]
PP053982	HVRI	Guinea-Bissau	Dulombi NP	11.812565	-14.396407	W	[27]
PP053983	HVRI	Guinea-Bissau	Dulombi NP	11.818034	-14.393708	W	[27]
PP053984	HVRI	Guinea-Bissau	Dulombi NP	11.818199	-14.394055	W	[27]
PP053985	HVRI	Guinea-Bissau	Dulombi NP	11.818353	-14.394057	W	[27]
PP053986	HVRI	Guinea-Bissau	Dulombi NP	11.818437	-14.393795	W	[27]
PP053987	HVRI	Guinea-Bissau	Dulombi NP	11.818366	-14.393722	W	[27]
PP053988	HVRI	Guinea-Bissau	Dulombi NP	11.818309	-14.393606	W	[27]

23 Haus, T.; Akom, E.; Agwanda, B.; Hofreiter, M.; Roos, C.; Zinner, D. Mitochondrial Diversity and Distribution of African Green Monkeys (*Chlorocebus* Gray, 1870). *Am. J. Primatol.* **2013**, *75*, 350–360. https://doi.org/10.1002/ajp.22113

24 Williams, D.M.; Almanza, S.M.; Sifuentes-Romero, I.; Detwiler, K.M. The History, Taxonomy, and Geographic Origins of an Introduced African Monkey in the Southeastern United States. *Primates* **2021**, *62*, 617–627. https://doi.org/10.1007/s10329-021-00890-1.

26 Gaubert, P.; Njiokou, F.; Olayemi, A.; Pagani, P.; Dufour, S.; Danquah, E.; Nutsuakor, M.E.K.; Ngua, G.; Missoup, A.; Tedesco, P.A.; et al. Bushmeat Genetics: Setting up a Reference Framework for the DNA Typing of African Forest Bushmeat. *Mol. Ecol. Resour.* **2015**, *15*, 633–651. https://doi.org/10.1111/1755-0998.12334.

27 Colmonero-Costeira, I.; Djaló, M.L.; Fernandes, N.; Borges, F.; Aleixo-Pais, I.; Gerini, F.; Costa, M., Minhós, T.; Ferreira da Silva, M.J. Improving Baseline Information on Over-Looked Generalists: Occurrence and Mitochondrial DNA Diversity of Campbell's (*Cercopithecus campbelli*) and Green Monkeys (*Chlorocebus sabaeus*) in Guinea-Bissau, West Africa. *Int. J. Primatol.* **2024**, *in press.*

33 Wang, Y. Molecular Polymorphisms for Phylogeny, Pedigree and Population Structure Studies. Ph.D. Thesis, University of Sydney School of Biological Sciences, Sydney, NSW, Australia, 2006.

43 Wertheim, J.O.; Worobey, M. A Challenge to the Ancient Origin of SIVagm Based on African Green Monkey Mitochondrial Genomes. *PLoS Pathog.* **2007**, *3*, 0866–0873. https://doi.org/10.1371/journal.ppat.0030095.

44 Guschanski, K.; Krause, J.; Sawyer, S.; Valente, L.M.; Bailey, S.; Finstermeier, K.; Sabin, R.; Gilissen, E.; Sonet, G.; Nagy, Z.T.; et al. Next-Generation Museomics Disentangles One of the Largest Primate Radiations. *Syst. Biol.* **2013**, *62*, 539–554. https://doi.org/10.1093/sysbio/syt018.

45 Dolotovskaya, S.; Torroba Bordallo, J.; Haus, T.; Noll, A.; Hofreiter, M.; Zinner, D.; Roos, C. Comparing Mitogenomic Timetrees for Two African Savannah Primate Genera (*Chlorocebus* and *Papio*). *Zool. J. Linn. Soc.* **2017**, *181*, 471–483. https://doi.org/10.1093/zoolinnean/zlx001.

46 Gossé, K.J.; Gonedelé-Bi, S.; Justy, F.; Chaber, A.L.; Kramoko, B.; Gaubert, P. DNA-Typing Surveillance of the Bushmeat in Côte d'Ivoire: A Multi-Faceted Tool for Wildlife Trade Management in West Africa. *Conserv. Genet.* **2022**, *23*, 1073–1088. https://doi.org/10.1007/s10592-022-01474-2.
